# Effects of adjunctive tirofiban during PCI on myocardial perfusion and ventricular function in myocardial infarction: a 3D echocardiography-based evaluation

**DOI:** 10.3389/fcvm.2026.1857341

**Published:** 2026-10-30

**Authors:** Xili Wang, Yingjuan Han, Chenxue Zhang

**Affiliations:** 1 Department of Ultrasound Medicine, Zhangjiakou First Hospital, Zhangjiakou, China; 2 Department of Cardiology, Zhangjiakou First Hospital, Zhangjiakou, China

**Keywords:** adjunctive tirofiban therapy, combined therapy, myocardial infarction, percutaneous coronary intervention, three-dimensional echocardiography, three-dimensional ultrasound, tirofiban

## Abstract

**Objective:**

This study aimed to evaluate the effects of adjunctive tirofiban during PCI on myocardial perfusion and ventricular functional recovery in patients with myocardial infarction using angiographic myocardial blush grade (MBG) and three-dimensional echocardiography (3DE).

**Methods:**

A retrospective review identified 114 eligible patients with ST-segment elevation myocardial infarction (STEMI) who underwent primary PCI at our hospital from March 2024 to May 2025, including 61 patients who received PCI alone and 53 who received PCI with adjunctive tirofiban. After 1:1 propensity score matching (PSM), 48 matched pairs (96 patients) were included in the final analysis. Myocardial perfusion was assessed using angiographic myocardial blush grade (MBG), whereas left ventricular ejection fraction (LVEF) was quantified from three-dimensional echocardiographic full-volume datasets using offline TomTec analysis. Major adverse cardiac events (MACE) were recorded during a 90-day follow-up after PCI and included cardiac death, recurrent myocardial infarction, hospitalization for worsening heart failure, and unplanned target-vessel revascularization.

**Results:**

The general baseline data of the two groups showed no statistically significant difference, indicating comparability (*P* > 0.05). Baseline MBG, LVEF, cTnI, SOD, and ET levels were also comparable between the two groups (all *P* > 0.05). Both groups showed significant changes from baseline after treatment, with greater improvements in these indicators observed in the treatment group (all *P* < 0.001 for between-group comparisons of changes). The myocardial blush grade (MBG) and left ventricular ejection fraction (LVEF) were significantly higher in the treatment group than in the control group (2.42 ± 1.02 vs. 1.58 ± 0.88 and 55.74 ± 3.12 vs. 48.89 ± 5.17, respectively; both *P* < 0.001). The cardiac troponin I (cTnI) level was significantly lower in the treatment group than in the control group (12.34 ± 1.76 vs. 16.73 ± 1.98, *P* < 0.001). The treatment group also showed a significantly higher superoxide dismutase (SOD) level (100.89 ± 3.21 vs. 90.11 ± 2.86, *P* < 0.001) and a significantly lower endothelin (ET) level (58.42 ± 1.65 vs. 77.24 ± 1.83, *P* < 0.001). During the 90-day follow-up, the incidence of MACE was significantly lower in the treatment group than in the control group (6/48, 12.50% vs. 17/48, 35.42%; *P* = 0.009).

**Conclusion:**

Adjunctive tirofiban during PCI was associated with greater improvements in myocardial perfusion and ventricular functional recovery than PCI alone. Three-dimensional echocardiography provided quantitative assessment of ventricular functional changes, while angiographic MBG provided complementary information on myocardial tissue perfusion. Given the single-center retrospective design, these findings should be interpreted cautiously and require confirmation in larger prospective studies.

## 1 Introduction

According to the Annual Report on Cardiovascular Health and Diseases in China 2024, the crude incidence of acute myocardial infarction among Chinese adults in 2023 was 87.6 per 100,000, with an age-standardized incidence rate of 93.9 per 100,000, and the incidence increased markedly with age (1). Globally, myocardial infarction (MI) remains a major cause of morbidity and mortality and continues to impose a substantial public health burden (2). Percutaneous coronary intervention (PCI), as the preferred reperfusion strategy for acute ST-segment elevation myocardial infarction (STEMI), can rapidly restore blood flow in the infarct-related artery and improve clinical outcomes (3–5). In patients with acute coronary syndrome (ACS) and multivessel coronary artery disease, complete revascularization is an important component of contemporary revascularization strategies, while coronary imaging modalities, including optical coherence tomography (OCT), intravascular ultrasound (IVUS), and coronary computed tomography angiography (CCTA), provide additional information on lesion morphology and can support lesion assessment and procedural planning in several clinical scenarios, including ACS (6–8). However, clinical practice has revealed that approximately 30%–50% of patients still exhibit microvascular obstruction (MVO) after PCI, leading to persistent myocardial ischemia, reperfusion injury, and ventricular remodeling, ultimately compromising long-term prognosis.

Tirofiban, a selective platelet glycoprotein IIb/IIIa receptor antagonist, inhibits platelet aggregation and has been shown to improve myocardial reperfusion when administered early in patients with ST-segment elevation myocardial infarction undergoing primary PCI (9). Subsequent analysis of the ON-TIME 2 program further showed that early pre-hospital high-bolus-dose tirofiban was associated with a lower 30-day major adverse cardiac event rate, without a clinically relevant increase in bleeding (10). Nevertheless, comprehensive evaluation after PCI requires assessment of both myocardial perfusion and ventricular function. Myocardial blush grade (MBG) is an angiographic indicator reflecting myocardial tissue-level perfusion, whereas three-dimensional echocardiography (3DE) enables full-volume acquisition and quantitative assessment of left ventricular function without relying on the geometric assumptions inherent to conventional two-dimensional echocardiography. In the present study, angiographic MBG was used to evaluate myocardial perfusion, while 3DE with offline post-processing was used to assess ventricular functional changes. This study evaluated adjunctive tirofiban during PCI using complementary angiographic and three-dimensional echocardiographic assessments to examine myocardial perfusion, cardiac functional recovery, oxidative stress status, and clinical prognosis in patients with myocardial infarction. By integrating angiographic perfusion assessment with three-dimensional echocardiographic evaluation of ventricular function, this study aimed to provide complementary evidence regarding short-term treatment response after PCI.

## 2 Objects and methods

### 2.1 Study subjects

This study was designed as a retrospective analysis of patients with ST-segment elevation myocardial infarction (STEMI) who underwent primary PCI at our hospital from March 2024 to May 2025. After screening according to the predefined inclusion and exclusion criteria, 114 eligible patients were identified. Patients were classified according to the PCI treatment strategy documented before the procedure: 61 patients scheduled to undergo PCI without a glycoprotein IIb/IIIa receptor inhibitor were assigned to the control group, whereas 53 patients scheduled to receive adjunctive tirofiban during PCI were assigned to the treatment group. Tirofiban use represented a planned adjunctive treatment strategy rather than bailout therapy initiated after the occurrence of slow-flow or no-reflow. To reduce baseline imbalance between the two groups, propensity scores were estimated using a binary logistic regression model incorporating age, sex, and baseline left ventricular ejection fraction (LVEF). Patients were matched at a 1:1 ratio using nearest-neighbor matching without replacement, with a caliper width of 0.20 standard deviations of the logit of the propensity score. Patients without an eligible match within the specified caliper were excluded. After matching, 48 matched pairs (96 patients) were retained for the final analysis, while 13 patients in the control group and 5 patients in the treatment group remained unmatched. Covariate balance was assessed using standardized mean differences (SMDs), with an absolute SMD < 0.10 considered indicative of adequate balance. For the propensity score-matched cohort, additional baseline and procedural characteristics were extracted from the medical records, including infarct location, culprit vessel, pre- and post-procedural Thrombolysis in Myocardial Infarction (TIMI) flow, number and mean diameter of implanted stents, door-to-balloon time, Killip class, and the post-PCI dual antiplatelet regimen. This retrospective study was approved by the Ethics Committee of Zhangjiakou First Hospital (Approval No. 2024XNLC-YWGC03) and was conducted in accordance with the Declaration of Helsinki. Owing to the retrospective design and the use of de-identified clinical data, the requirement for informed consent was waived by the Ethics Committee. Privacy safeguards included: anonymization of data prior to collection by removing personally identifiable information (e.g., names, ID numbers); establishment of a tiered access control system restricting full dataset access to authorized researchers only; and presentation of aggregated clinical data in publications to eliminate individual identification risks.

### 2.2 Inclusion and exclusion criteria

Inclusion criteria required patients to meet the following conditions: first-time clinically diagnosed ST-segment elevation myocardial infarction (STEMI) with confirmed left ventricular dysfunction on echocardiography (11–13); treatment with primary PCI; no history of drug allergies or adverse reactions to treatment-related medications; and complete clinical records.

Exclusion criteria specified the following: patients with severe hepatic or renal dysfunction; prior history of myocardial infarction; previous cardiac surgical procedures; bleeding tendencies or coagulation disorders; and severe vascular calcification.

### 2.3 Treatment protocols

All patients received standard antiplatelet therapy with aspirin and clopidogrel before PCI, together with procedural anticoagulation using unfractionated heparin according to the institutional PCI protocol. Routine systemic fibrinolytic therapy was not administered in this primary PCI cohort. The decision to use adjunctive tirofiban was made by the treating interventional cardiologist before PCI as part of the planned antiplatelet strategy, with the aim of providing more intensive platelet inhibition and reducing thrombotic microembolization during primary PCI. Tirofiban administration was not initiated as bailout treatment in response to established intra-procedural slow-flow or no-reflow, and the occurrence of no-reflow was not used to determine treatment-group assignment. No prespecified thrombus-burden threshold was used as a mandatory trigger for tirofiban administration. Patients in the control group underwent PCI without administration of a glycoprotein IIb/IIIa receptor inhibitor. In the treatment group, tirofiban was administered intracoronarily at a bolus dose of 10 μg/kg through the guiding catheter during PCI, followed immediately by a continuous intravenous infusion at 0.15 μg/kg/min for 18 h. After PCI, both groups received standard secondary-prevention therapy, including aspirin 100 mg once daily and clopidogrel 75 mg once daily, unless contraindicated. Unfractionated heparin was used for periprocedural anticoagulation only, and routine post-procedural parenteral anticoagulation was not included in the treatment protocol for either group.

### 2.4 Three-dimensional echocardiographic and angiographic assessment

Three-dimensional echocardiography was performed using a GE Vivid E95 ultrasound system equipped with a 4 V probe. Left ventricular full-volume datasets were acquired before PCI and on postoperative day 7 and were stored for subsequent offline analysis using TomTec software. LVEF was quantified from the three-dimensional echocardiographic datasets by an experienced echocardiographer blinded to treatment allocation.

MBG was assessed separately from coronary angiograms obtained before PCI and from the final angiogram after PCI rather than from the three-dimensional echocardiographic images. MBG was graded on a 0–3 scale: grade 0, no myocardial blush; grade 1, minimal myocardial blush; grade 2, moderate myocardial blush but less than that observed in a non-infarct-related myocardial territory; and grade 3, normal myocardial blush comparable to that of a non-infarct-related territory. Two experienced interventional cardiologists independently reviewed the de-identified angiograms while blinded to treatment allocation and the laboratory and echocardiographic results. Disagreements were resolved by consensus. Inter-reader agreement for MBG grading was evaluated using weighted Cohen's *κ*.

### 2.5 Laboratory indicator assays

Venous blood samples (5 mL each) were collected from fasting patients before PCI and on postoperative day 7. After centrifugation at 3,500 rpm for 5 min (radius: 10.4 cm), serum superoxide dismutase (SOD) activity was measured via chemiluminescent immunoassay, while endothelin (ET) and cardiac troponin I (cTnI) concentrations were determined by enzyme-linked immunosorbent assay (ELISA). Reagent kits were sourced from Nanjing Jiancheng Bioengineering Institute and Wuhan Boster Biological Technology Co., Ltd.

### 2.6 Follow-up, MACE, and safety assessment

Clinical follow-up was initiated on the date of PCI and continued for 90 days. Follow-up information was obtained from outpatient records, rehospitalization records, and structured telephone follow-up at approximately 30 and 90 days after PCI. Major adverse cardiac events (MACE) were defined as a composite of cardiac death, recurrent myocardial infarction, hospitalization for worsening heart failure, and unplanned target-vessel revascularization. Recurrent myocardial infarction was defined as a new myocardial infarction occurring after the index PCI and documented by compatible clinical symptoms, electrocardiographic changes, and/or a new rise in cardiac biomarkers. Heart failure events were counted only when worsening heart failure resulted in hospital admission. Unplanned target-vessel revascularization referred to repeat PCI or coronary artery bypass grafting of the treated target vessel because of recurrent ischemia. For the composite MACE endpoint, each patient was counted once regardless of whether more than one event occurred during follow-up. No patients in the propensity score-matched cohort were lost to the 90-day follow-up.

Treatment-related safety events were also reviewed from the index hospitalization through the 90-day follow-up using inpatient records, outpatient records, and rehospitalization data. Safety outcomes included bleeding events, thrombocytopenia, blood transfusion, access-site hematoma, intracranial hemorrhage, and other clinically documented treatment-related adverse reactions. Bleeding events were classified according to the Bleeding Academic Research Consortium (BARC) criteria, with BARC types 1–2 considered minor bleeding and BARC types 3–5 considered major bleeding. Thrombocytopenia was defined as a platelet count <100 × 10^9^/L or a decrease of >50% from the baseline platelet count.

### 2.7 Statistical analysis

Image processing was performed using GraphPad Prism 8, and statistical analyses were conducted using SPSS version 26.0. Because this was a retrospective study in which all eligible patients treated during the predefined study period were screened for inclusion, no *a priori* sample size calculation was performed. A sensitivity power analysis was subsequently conducted using G*Power version 3.1.9.7. With 48 patients per group in the propensity score-matched cohort, a two-sided *α* of 0.05, and 80% statistical power, the available sample size was sufficient to detect a standardized between-group effect size (Cohen's d) of approximately 0.58 for continuous outcomes.

Continuous variables were expressed as mean ± standard deviation. Independent-samples t-tests were used for between-group comparisons, while paired t-tests were used for within-group comparisons between baseline and post-treatment values. Changes from baseline (*Δ*), calculated as the post-treatment value minus the baseline value, were compared between groups using independent-samples t-tests. Categorical variables were presented as *n* (%) and compared using Pearson's *χ*^2^ test when the expected cell frequencies were adequate; Fisher's exact test was used when any expected cell frequency was <5. The comparison of 90-day MACE incidence between the two groups was performed using Pearson's *χ*^2^ test because all expected cell frequencies exceeded 5. Inter-reader agreement for MBG grading was assessed using weighted Cohen's *κ*.

To account for multiple outcome testing, the family-wise error rate across the six principal outcome domains (MBG, LVEF, cTnI, SOD, ET, and 90-day MACE) was controlled using the Holm–Bonferroni procedure. For continuous outcomes measured before and after treatment, the between-group comparison of change from baseline was considered the principal treatment-effect test, whereas within-group comparisons and post-treatment between-group comparisons were regarded as supportive analyses. Two-sided *P* values <0.05 after multiplicity adjustment were considered statistically significant.

## 3 Results

### 3.1 Baseline and procedural characteristics

Before PSM, 114 eligible patients were identified, including 61 patients in the control group and 53 patients in the treatment group. Following 1:1 nearest-neighbor matching, 48 matched pairs were retained for analysis. The SMDs for age, sex, and baseline LVEF decreased from 0.298, 0.171, and 0.484 before matching to 0.035, 0.042, and 0.052 after matching, respectively. All post-matching SMDs were < 0.10, indicating adequate balance of the matching covariates. After propensity score matching, 48 patients were included in each group. The control and treatment groups were comparable in age, sex, baseline LVEF, hypertension, diabetes, and hyperlipidemia (all *P* > 0.05). No significant between-group differences were observed in infarct location (*χ*^2^ = 0.500, *P* = 0.919), culprit vessel (*χ*^2^ = 0.201, *P* = 0.904), pre-procedural TIMI flow (*χ*^2^ = 0.515, *P* = 0.473), post-procedural TIMI flow (*χ*^2^ = 0.447, *P* = 0.504), or Killip class (*χ*^2^ = 0.468, *P* = 0.791). The number of implanted stents (1.35 ± 0.56 vs. 1.31 ± 0.51, *P* = 0.715), mean stent diameter (3.02 ± 0.34 vs. 3.05 ± 0.32 mm, *P* = 0.657), and door-to-balloon time (74.80 ± 14.60 vs. 72.90 ± 13.80 min, *P* = 0.514) were also comparable between the control and treatment groups. All patients in both groups received the same post-PCI dual antiplatelet regimen consisting of aspirin 100 mg once daily plus clopidogrel 75 mg once daily. The detailed baseline and procedural characteristics are presented in Table 1.

**Table 1:** Comparison of baseline and procedural characteristics between the two groups.

Variable	Control group (*n* = 48)	Treatment Group (*n* = 48)	*t/x^2^*	*P*
Gender, *n* (%)			0.042	0.838
Male	25 (52.08)	24 (50.00)	—	—
Female	23 (47.92)	24 (50.00)	—	—
Age (years)	53.89 ± 5.22	53.71 ± 5.17	0.170	0.866
Baseline LVEF (%)	43.82 ± 4.91	44.07 ± 4.76	−0.253	0.801
Hypertension, *n* (%)	11 (22.92)	12 (25.00)	0.057	0.811
Diabetes, *n* (%)	8 (16.67)	6 (12.50)	0.334	0.563
Hyperlipidemia, *n* (%)	10 (20.83)	11 (22.92)	0.061	0.805
Infarct location, *n* (%)			0.500	0.919
Anterior	23 (47.92)	20 (41.67)	—	—
Inferior	14 (29.17)	17 (35.42)	—	—
Lateral	6 (12.50)	6 (12.50)	—	—
Posterior	5 (10.42)	5 (10.42)	—	—
Culprit vessel, *n* (%)			0.201	0.904
LAD	25 (52.08)	23 (47.92)	—	—
RCA	16 (33.33)	18 (37.50)	—	—
LCX	7 (14.58)	7 (14.58)	—	—
Pre-procedural TIMI flow, *n* (%)			0.515	0.473
TIMI 0–1	38 (79.17)	35 (72.92)	—	—
TIMI 2–3	10 (20.83)	13 (27.08)	—	—
Post-procedural TIMI flow, *n* (%)			0.447	0.504
TIMI 3	42 (87.50)	44 (91.67)	—	—
TIMI <3	6 (12.50)	4 (8.33)	—	—
Number of implanted stents	1.35 ± 0.56	1.31 ± 0.51	0.366	0.715
Mean stent diameter (mm)	3.02 ± 0.34	3.05 ± 0.32	−0.445	0.657
Door-to-balloon time (min)	74.80 ± 14.60	72.90 ± 13.80	0.655	0.514
Killip class, *n* (%)			0.468	0.791
I	27 (56.25)	30 (62.50)	—	—
II	16 (33.33)	13 (27.08)	—	—
III–IV	5 (10.42)	5 (10.42)	—	—
Post-PCI DAPT: aspirin+clopidogrel, *n* (%)	48 (100.00)	48 (100.00)	—	—

Continuous variables are presented as mean ± standard deviation and were compared using independent-samples t-tests. Categorical variables are presented as *n* (%) and were compared using Pearson's *χ*^2^ tests. Overall *χ*^2^ statistics and *P* values are reported for variables with multiple categories. TIMI, Thrombolysis in Myocardial Infarction; LAD, left anterior descending artery; RCA, right coronary artery; LCX, left circumflex artery; DAPT, dual antiplatelet therapy. Statistical comparison of the post-PCI DAPT regimen was not performed because all patients received the same regimen.

### 3.2 Cardiac function indicators

Inter-reader agreement for angiographic MBG grading was high (weighted Cohen's *κ* = 0.86). Before PCI, angiographic MBG was comparable between the control and treatment groups (0.76 ± 0.63 vs. 0.79 ± 0.65, *P* = 0.819), and baseline LVEF was also comparable (43.82 ± 4.91% vs. 44.07 ± 4.76%, *P* = 0.801). Following PCI, final angiographic MBG increased significantly in both groups, while LVEF measured on postoperative day 7 also increased significantly from baseline. MBG increased by 0.82 ± 0.66 in the control group and 1.63 ± 0.71 in the treatment group, corresponding to a between-group difference in change of 0.81 (95% CI: 0.53–1.09; *P* < 0.001). LVEF increased by 5.07 ± 3.51% and 11.67 ± 3.42% in the control and treatment groups, respectively, with a between-group difference in change of 6.60% (95% CI: 5.20–8.00; *P* < 0.001). At the post-treatment assessment, the mean between-group difference was 0.84 (95% CI: 0.45–1.23; *P* < 0.001) for MBG and 6.85% (95% CI: 5.12–8.58; *P* < 0.001) for LVEF. See Figure 1.

**Figure 1:**
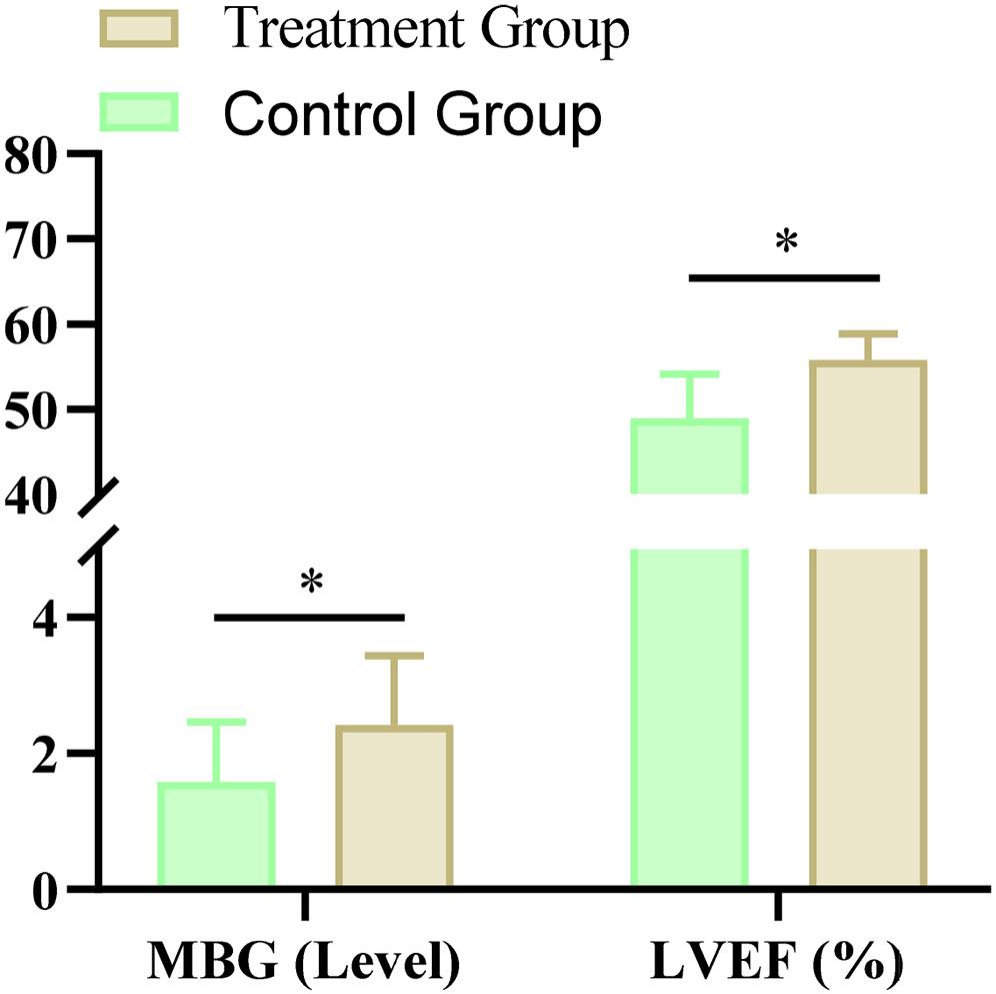
Comparison of Post-PCI MBG and Post-treatment LVEF Between the two Groups. * indicates statistically significant differences between groups (*P* < 0.05).

### 3.3 Myocardial injury

Baseline cTnI levels were comparable between the control and treatment groups (24.91 ± 3.22 vs. 24.57 ± 3.18 ng/mL, *P* = 0.604). After treatment, cTnI levels decreased significantly from baseline in both groups. The mean changes were −8.18 ± 2.84 ng/mL in the control group and −12.23 ± 2.71 ng/mL in the treatment group, corresponding to a between-group difference in change of −4.05 ng/mL (95% CI: −5.17 to −2.93; *P* < 0.001). At the post-treatment assessment, the cTnI level was significantly lower in the treatment group than in the control group, with a mean between-group difference of −4.39 ng/mL (95% CI: −5.15 to −3.63; *P* < 0.001). See Figure 2.

**Figure 2:**
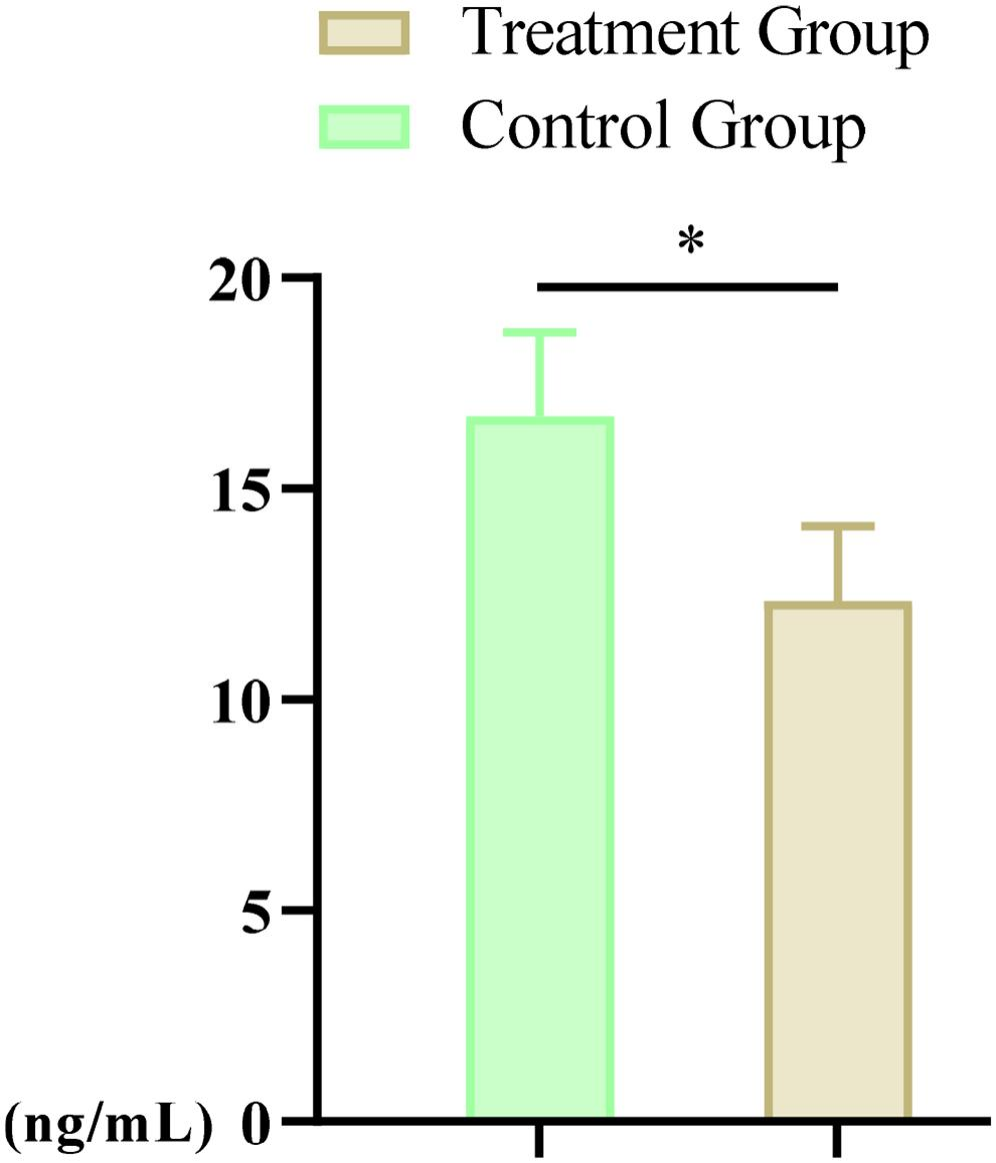
Comparison of cTnI Levels Between two Groups. * indicates statistically significant differences between groups (*P* < 0.05).

### 3.4 Oxidative stress

At baseline, SOD and ET levels were comparable between the control and treatment groups (SOD: 78.66 ± 4.15 vs. 79.02 ± 4.08 U/L, *P* = 0.669; ET: 89.75 ± 3.21 vs. 89.12 ± 3.34 ng/L, *P* = 0.348). After treatment, SOD increased and ET decreased significantly from baseline in both groups. The mean increase in SOD was 11.45 ± 4.06 U/L in the control group and 21.87 ± 4.21 U/L in the treatment group, corresponding to a between-group difference in change of 10.42 U/L (95% CI: 8.74–12.10; *P* < 0.001). The mean reduction in ET was −12.51 ± 3.63 ng/L in the control group and −30.70 ± 3.91 ng/L in the treatment group, with a between-group difference in change of −18.19 ng/L (95% CI: −19.72 to −16.66; *P* < 0.001). At the post-treatment assessment, the mean between-group difference was 10.78 U/L (95% CI: 9.55–12.01; *P* < 0.001) for SOD and −18.82 ng/L (95% CI: −19.53 to −18.11; *P* < 0.001) for ET. See Figure 3.

**Figure 3:**
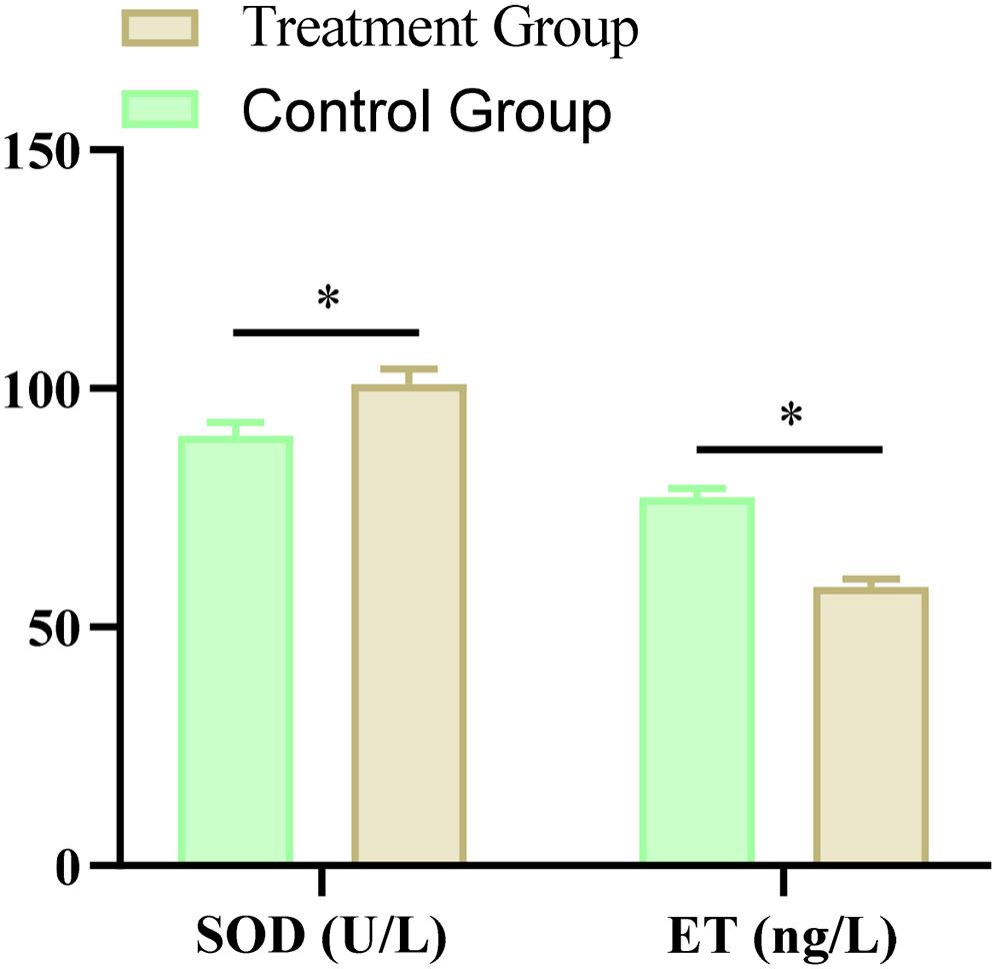
Comparison of SOD and ET Levels Between two Groups. * indicates statistically significant differences between groups (*P* < 0.05).

### 3.5 Major adverse cardiac events and safety outcomes

All 96 patients in the propensity score-matched cohort completed the 90-day follow-up. MACE occurred in 17 of 48 patients (35.42%) in the control group and in 6 of 48 patients (12.50%) in the treatment group. Based on the first qualifying MACE event, the control group included 2 cardiac deaths, 4 recurrent myocardial infarctions, 8 hospitalizations for worsening heart failure, and 3 unplanned target-vessel revascularizations, whereas the treatment group included 1 cardiac death, 1 recurrent myocardial infarction, 3 hospitalizations for worsening heart failure, and 1 unplanned target-vessel revascularization. Pearson's *χ*^2^ test demonstrated that the overall 90-day MACE incidence was significantly lower in the treatment group than in the control group (12.50% vs. 35.42%; *χ*^2^ = 6.918, *P* = 0.009), corresponding to a relative risk of 0.35 (95% CI: 0.15–0.82). After Holm–Bonferroni correction across the six principal outcome domains, the between-group changes in MBG, LVEF, cTnI, SOD, and ET remained statistically significant (all adjusted *P* < 0.001), and the difference in 90-day MACE incidence also remained significant (adjusted *P* = 0.009). See Figure 4. Regarding treatment-related safety events, bleeding occurred in 3 patients (6.25%) in the control group and 6 patients (12.50%) in the treatment group (*P* = 0.486). BARC type 1–2 bleeding occurred in 3 patients (6.25%) and 5 patients (10.42%), respectively (*P* = 0.714), while BARC type 3–5 bleeding occurred in no patients in the control group and 1 patient (2.08%) in the treatment group (*P* = 1.000). Thrombocytopenia was observed in 1 patient (2.08%) in the control group and 2 patients (4.17%) in the treatment group (*P* = 1.000). No patient in the control group required blood transfusion, whereas 1 patient (2.08%) in the treatment group received transfusion (*P* = 1.000). Access-site hematoma occurred in 2 patients (4.17%) and 3 patients (6.25%) in the control and treatment groups, respectively (*P* = 1.000). No intracranial hemorrhage, fatal bleeding, or severe allergic reaction occurred in either group. Fisher's exact test showed no statistically significant between-group differences in the recorded treatment-related safety events (all *P* > 0.05).

**Figure 4:**
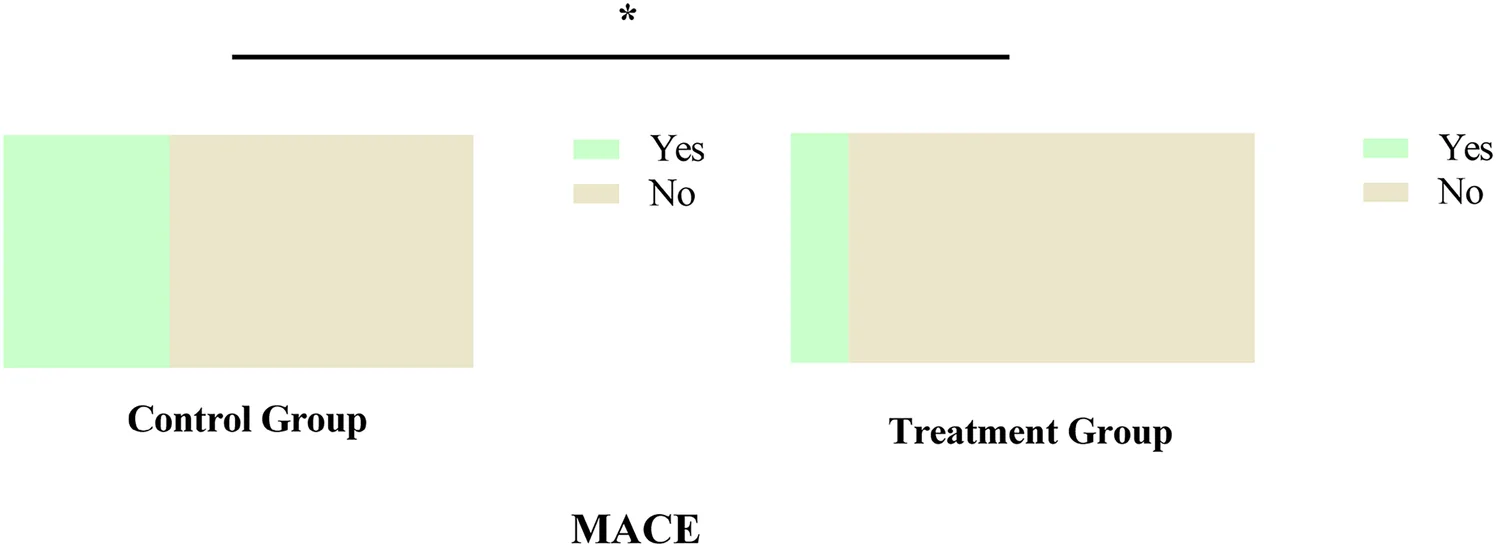
Comparison of 90-day MACE Incidence Between the two Groups. * indicates statistically significant differences between groups (*P* < 0.05).

## 4 Discussion

Following acute myocardial infarction (AMI), myocardial cells undergo damage and death. Infiltration of inflammatory cells disrupts the collagen scaffold structure, leading to thinning and dilation of the infarcted myocardium, which subsequently accelerates left ventricular remodeling. This left ventricular remodeling induces ventricular wall thinning, dilation, and morphological distortion, ultimately impairing cardiac pumping function and resulting in severe clinical outcomes such as heart failure, arrhythmias, and even death (14, 15). Currently, two-dimensional echocardiography using the Simpson method and M-mode ultrasound are commonly employed noninvasive techniques for ventricular assessment. While these methods demonstrate acceptable accuracy and reproducibility in measuring left ventricular function when ventricular geometry is relatively preserved, they remain limited by their reliance on geometric assumptions of the left ventricle. In the presence of abnormal or asymmetric ventricular geometry, including that associated with myocardial infarction and regional wall-motion abnormalities, such assumptions may reduce the accuracy of left ventricular volume measurements (16–18). Compared to conventional ultrasound methods, previous studies have demonstrated the value of three-dimensional echocardiography (3DE) for quantitative assessment of left ventricular geometry and function after myocardial infarction (19–21).

This study evaluated the effects of adjunctive tirofiban during PCI using complementary angiographic assessment of myocardial perfusion and three-dimensional echocardiographic assessment of ventricular function. The post-matching comparisons showed adequate balance in the prespecified matching covariates, with all corresponding SMDs below 0.10. The use of PSM therefore improved comparability between the two groups with respect to measured baseline characteristics, although residual confounding from unmeasured or incompletely measured factors cannot be excluded because of the retrospective study design.

In terms of cardiac function indicators, the significantly higher myocardial blush grade (MBG) and left ventricular ejection fraction (LVEF) observed in the treatment group suggest potential benefits associated with adjunctive tirofiban during PCI in improving myocardial perfusion and cardiac function. As a platelet glycoprotein IIb/IIIa receptor antagonist, tirofiban effectively inhibits platelet aggregation by blocking fibrinogen binding to platelet receptors, thereby reducing coronary microthrombus formation and improving microcirculatory perfusion in myocardial tissue. This improvement in tissue-level perfusion, reflected by the higher angiographic MBG observed after PCI, suggests improved myocardial reperfusion quality. Concurrently, the marked increase in LVEF further confirms substantive recovery of cardiac function. Previous studies have shown that three-dimensional echocardiography and three-dimensional speckle-tracking echocardiography can quantitatively characterize left ventricular geometry and myocardial deformation after acute myocardial infarction, and that several three-dimensional strain parameters are associated with subsequent left ventricular remodeling (19–21). Notably, the concurrent elevation of MBG and LVEF forms a complete evidence chain from microcirculatory perfusion to macroscopic cardiac function, revealing the dual advantages of combined therapy in improving myocardial perfusion and cardiac function recovery.

The significant reduction in cardiac troponin I (cTnI) levels, a myocardial injury biomarker, provides molecular-level evidence for the protective effects of combined therapy on myocardial cells. As a specific marker of myocardial cell damage, cTnI levels directly reflect the extent of myocardial injury (22–24). The marked decrease in cTnI levels in the treatment group indicates that tirofiban combined with PCI effectively reduces myocardial cell necrosis and apoptosis, likely attributable to tirofiban's potent inhibition of platelet activation. By reducing coronary thrombus burden and lowering distal embolization risk, tirofiban alleviates myocardial ischemic injury. Additionally, tirofiban may further reduce myocardial damage through mechanisms such as modulating inflammatory responses and decreasing oxidative stress, with these multipathway protective effects quantitatively reflected in the reduced cTnI levels.

Changes in oxidative stress indicators, superoxide dismutase (SOD) and endothelin (ET), reveal the regulatory effects of adjunctive tirofiban during PCI on myocardial oxidative stress status. Elevated SOD activity, a key antioxidant enzyme, reflects enhanced systemic antioxidant capacity, while reduced ET levels, a potent vasoconstrictor peptide secreted by vascular endothelial cells, suggest improved vascular endothelial function (25–27). The significant increases in SOD and decreases in ET levels in the treatment group indicate that tirofiban combined with PCI modulates oxidative stress balance, reduces myocardial oxidative damage, and improves vascular endothelial function, thereby promoting coronary vasodilation and blood flow restoration. This dual regulation of oxidative stress and endothelial function may occur through pathways such as reducing free radical generation, enhancing antioxidant defenses, and improving endothelial cell function, creating a favorable microenvironment for myocardial cell protection and cardiac function recovery (28, 29).

During the 90-day follow-up, the incidence of MACE was lower in the treatment group than in the control group. In the present study, MACE was defined as a composite of cardiac death, recurrent myocardial infarction, hospitalization for worsening heart failure, and unplanned target-vessel revascularization. The relatively high 90-day MACE incidence observed in the control group (35.42%) should therefore be interpreted in the context of this broad composite endpoint rather than as a mortality rate alone. In addition, the study population consisted of patients with myocardial infarction and baseline left ventricular dysfunction, which may have contributed to a relatively high short-term event burden. The difference in MACE incidence may be related to several potential mechanisms: adjunctive tirofiban may reduce thrombotic complications by inhibiting platelet aggregation, while improved myocardial perfusion and ventricular functional recovery may contribute to a lower risk of recurrent ischemic events and worsening heart failure (30–34). Nevertheless, because this was a retrospective study with a limited sample size, the observed difference in 90-day MACE should be interpreted cautiously and requires confirmation in larger prospective studies. With regard to safety, bleeding events were numerically more frequent in patients receiving adjunctive tirofiban, but the differences in overall bleeding, major bleeding, thrombocytopenia, and transfusion were not statistically significant between the two groups. No intracranial hemorrhage or fatal bleeding was observed. Nevertheless, given the relatively limited sample size, the present study may not have been sufficiently powered to detect uncommon but clinically important bleeding complications, and the safety findings should therefore be interpreted cautiously.

The potential influence of post-procedural anticoagulation on clinical outcomes after primary PCI should also be considered. Zhou et al. reported that, among patients with ST-segment elevation myocardial infarction complicated by cardiogenic shock who underwent primary PCI, post-procedural anticoagulation was associated with a lower risk of in-hospital all-cause mortality (14.9% vs. 30.3%; adjusted HR, 0.60; 95% CI, 0.37–0.97; *P* = 0.037), without a statistically significant increase in major bleeding (35, 36). These findings suggest that the intensity and timing of antithrombotic therapy after PCI may influence early clinical outcomes in selected high-risk patients. However, direct comparison with the present study should be made cautiously because the study by Zhou et al. specifically included patients with cardiogenic shock and evaluated post-procedural anticoagulation, whereas our study evaluated planned adjunctive tirofiban during PCI and did not include routine post-procedural parenteral anticoagulation. Therefore, the lower 90-day MACE incidence observed in our treatment group should not be attributed to post-procedural anticoagulation.

## 5 Limitations

Although this study observed potential benefits associated with adjunctive tirofiban during PCI and demonstrated the utility of three-dimensional echocardiography for ventricular functional assessment, several limitations warrant consideration. First, this was a single-center retrospective study with a relatively limited sample size of 96 patients (48 patients in each group after propensity score matching [PSM]), which may be subject to selection bias and may have limited statistical power for detecting small treatment effects or infrequent clinical events. Despite the improved balance in measured baseline covariates after PSM, the retrospective design still makes it difficult to fully control for unknown confounding factors, such as differences in patient medication adherence and postoperative rehabilitation management, all of which may affect the generalizability of the results. In addition, although propensity score matching was performed to improve baseline comparability between the two groups, subsequent between-group analyses were conducted using independent-samples methods rather than statistical approaches that explicitly accounted for the matched-pair structure, which may have affected the consistency and robustness of the estimates. In addition, because the decision to use adjunctive tirofiban was made by the treating interventional cardiologist rather than through randomized treatment allocation, residual treatment-selection bias cannot be completely excluded. Moreover, because the present study compared PCI alone with PCI plus tirofiban and did not include a tirofiban-alone arm or a factorial design, the findings should be interpreted as reflecting the potential additional benefit of adjunctive tirofiban rather than evidence of a synergistic interaction between tirofiban and PCI. Second, clinical outcome follow-up in the present study was limited to 90 days after PCI. Although this period permitted assessment of short-term MACE, it was insufficient to evaluate longer-term outcomes such as sustained ventricular remodeling, chronic heart failure, recurrent ischemic events beyond 90 days, and long-term survival. Ventricular remodeling after myocardial infarction is a dynamic process, and short-term improvements in cardiac function do not necessarily translate into durable long-term benefits. Future studies should extend follow-up to at least 6–12 months and incorporate high-resolution techniques such as cardiac magnetic resonance imaging to evaluate longer-term changes in ventricular remodeling, myocardial fibrosis, and microcirculatory perfusion. Third, this study only focused on two oxidative stress indicators, SOD and ET, whereas myocardial ischemia-reperfusion injury involves complex multidimensional pathological mechanisms, including inflammatory responses, apoptosis, and mitochondrial dysfunction. Future research could expand the biomarker profile to include oxidative stress indicators such as NADPH oxidase and 8-hydroxydeoxyguanosine, as well as inflammatory factors like IL-6 and TNF-α. Combined with single-cell sequencing technology, this would reveal the interactive mechanisms among myocardial cells, endothelial cells, and immune cells during combined treatment, thereby more deeply elucidating the molecular basis of its myocardial protective effects. Although three-dimensional echocardiography enables quantitative assessment of ventricular geometry and function, myocardial perfusion in the present study was evaluated using angiographic MBG. Future studies incorporating complementary multimodal imaging techniques may provide a more comprehensive assessment of both ventricular remodeling and myocardial microcirculatory perfusion. Simultaneously, the development of artificial intelligence-assisted three-dimensional ultrasound image analysis algorithms could enable automated quantification of myocardial remodeling parameters, reduce human measurement errors, and improve research efficiency and result reliability.

## 6 Conclusion

In summary, adjunctive tirofiban during PCI was associated with improvements in myocardial perfusion and ventricular functional recovery. Three-dimensional echocardiography provided quantitative assessment of ventricular function, whereas angiographic MBG provided complementary assessment of myocardial tissue perfusion. However, this study still has certain limitations, such as a relatively small sample size and a short study period. Future research should further validate the long-term efficacy and safety of combined treatment through multicenter, large-sample randomized controlled trials. Meanwhile, this study offers new insights and methods for precision treatment of myocardial infarction, with the potential to drive further advancements in the field of cardiovascular disease treatment.

## Data Availability

The original contributions presented in the study are included in the article/Supplementary Material, further inquiries can be directed to the corresponding author.
